# Pyrethroid Resistance Aggravation in Ugandan Malaria Vectors Is Reducing Bednet Efficacy

**DOI:** 10.3390/pathogens10040415

**Published:** 2021-04-01

**Authors:** Magellan Tchouakui, Leon M. J. Mugenzi, Benjamin D. Menze, Jude N. T. Khaukha, Williams Tchapga, Micareme Tchoupo, Murielle J. Wondji, Charles S. Wondji

**Affiliations:** 1 Medical Entomology Department, Centre for Research in Infectious Diseases (CRID), Yaoundé, Cameroon; leon.mugenzi@crid-cam.net (L.M.J.M.); benjamin.menze@crid-cam.net (B.D.M.); williams.tchapga@crid-cam.net (W.T.); micareme.tchoupo@crid-cam.net (M.T.); murielle.wondji@lstmed.ac.uk (M.J.W.); 2 Department of Biochemistry and Molecular Biology, Faculty of Science University of Buea, Buea, Cameroon; 3 Entomology department, Uganda Virus Research Institute (UVRI), Entebbe, Uganda; judet21@gmail.com; 4 Vector Biology Department, Liverpool School of Tropical Medicine, Pembroke Place, Liverpool L3 5QA, UK

**Keywords:** malaria, resistance escalation, vector control, *An. funestus*, Uganda, metabolic resistance, cytochrome P450, *CYP9K1*

## Abstract

Monitoring cases of insecticide resistance aggravation and the effect on the efficacy of control tools is crucial for successful malaria control. In this study, the resistance intensity of major malaria vectors from Uganda was characterised and its impact on the performance of various insecticide-treated nets elucidated. High intensity of resistance to the discriminating concentration (DC), 5× DC, and 10× DC of pyrethroids was observed in both *Anopheles funestus* and *Anopheles gambiae* in Mayuge and Busia leading to significant reduced performance of long-lasting insecticidal nets (LLINs) including the piperonyl butoxide (PBO)-based nets (Olyset Plus). Molecular analysis revealed significant over-expression of cytochrome P450 genes (*CYP9K1* and *CYP6P9a*/*b*). However, the expression of these genes was not associated with resistance escalation as no difference was observed in the level of expression in mosquitoes resistant to 5× DC and 10× DC compared to 1× DC suggesting that other resistance mechanisms are involved. Such high intensity of pyrethroid resistance in Uganda could have terrible consequences on the effectiveness of insecticide-based interventions and urgent action should be taken to prevent the spread of super-resistance in malaria vectors.

## 1. Introduction

Malaria remains a major public health problem, with about 229 million cases and 409,000 deaths recorded in 2019 mainly in children under five years [1]. Uganda accounts for the sixth-highest malaria burden in Africa with approximately 14 million cases recorded in 2017 leading to 10,500 annual deaths [2]. Both long-lasting insecticidal nets (LLINs) and indoor residual spraying (IRS) are the key prevention measures in this country as in many other African countries. Unfortunately, these interventions are facing the challenge of insecticide resistance occurring in the main malaria vectors.

Increasing reports of widespread resistance to pyrethroids are being made for the major malaria vectors Africa-wide [3,4,5,6]. Furthermore, increased resistance levels are also been reported and has been suggested to potentially present a greater risk of control failure [7]. For example, a tremendous loss of performance of pyrethroid-only and piperonyl butoxide (PBO)-based nets (nets that contains the synergist PBO and the insecticide) in a resistant population of *An. funestus* s.s. was shown recently in southern Mozambique (Southern Africa) [4,8]. Similarly, a study in Western Africa (Burkina Faso revealed that increased resistance in *An. gambiae* was associated with the low efficacy of pyrethroid-only nets [9]. In Chad (Central Africa), a study conducted in *An. coluzzii* pointed to similar reduced efficacy of standard nets and the second generation net Olyset plus due to a high level of resistance [6]. Therefore, unless such super-resistance is managed, recent gains in reducing malaria transmission could be lost with terrible consequences. Monitoring cases of aggravation of resistance intensity and its impact on the efficacy of control tools is therefore crucial to predict consequences of resistance and to implement suitable control measures.

However, standardised methods have not always been used to monitor the extent of resistance in the field preventing to accurately quantify the resistance intensity and to establish the molecular drivers of such aggravation. Lack of routine quantification of resistance aggravation has also prevented to establish the impact of resistance on the efficacy of insecticide-based tools such as long lasting insecticidal nets (LLINs).

In that order, to harmonise the estimation of resistance levels, WHO issued a protocol relying on the testing of field populations with different concentration of insecticides at (1×, 5× and 10× concentration) [10]. This system now enables to better quantify the extent of resistance escalation in the field populations of mosquitoes and also facilitates the exploration of mechanisms driving that aggravation. Furthermore, it could also allow to evaluate the influence of super-resistance on the performance of insecticide-treated tools. Pyrethroid-based insecticides have been used in the agricultural sector in Uganda since the early 1990s and from 2000 to 2010 in IRS programs [2]. In 2014, a universal LLIN coverage campaign (one LLIN per two people) was attempted in the country and more than 22 million LLINs were distributed. IRS was implemented later from 2007 to 2014 in 10 districts in northern Uganda supporting by USAID/PMI and the Department for International Development (DFID). This strategy was extended later in other new highly malaria-endemic districts (14 in total) in the northern and eastern parts of the country [2]. This control approach coupled with the massive use of agricultural pesticides exert insecticidal pressure on local malaria vectors, which may accelerate the development and spread of insecticide resistance. Such a situation offers an ideal opportunity to quantify resistance intensity and its impact on vector tools to help improve resistance management strategies.

Here we have used the WHO method for resistance intensity measurement, to thoroughly and comparatively measure the extent of resistance escalation in two major malaria vectors, explore the potential molecular drivers behind such aggravation and establish the effect of such super-resistance on the efficacy of LLINs. Our study reveals a high intensity of resistance to pyrethroids in both species from the two locations with an extensive reduced performance of all pyrethroid-based LLINs tested, including PBO-based LLINs. This resistance escalation was associated with a high expression of key cytochrome P450 genes in *An. funestus* s.s. and a fixation of the 1014S-Kdr-E resistance allele in *An gambiae*.

## 2. Results

### 2.1. Sampling

In total, 25 houses were surveyed in Busia (Bumanji) and 35 in Mayuge (Bubbalya) (Table 1). The predominant type of house was rectangular with a corrugated iron roof in Busia (52%) followed by both circular hut with a thatched roof (24%) and rectangular hut with a thatched roof (24%). However, in Mayuge, the predominant type was rectangular huts with thatched roof (48.6%) followed by rectangular huts with a corrugated iron roof (40%) with only few (11.4%) circular huts with a thatched roof (Table S1). The main vector control measure in the collection sites was impregnated bed net. Table 1 summarises the brand of bed nets used in both collection sites. Out of the 25 houses surveyed in Busia, 23 had at least one bed net corresponding to the coverage rate of 92%. Of the 89 inhabitants living in those houses, only 53 (59%) were really sleeping under a net. The bed net coverage rate was almost the same in Mayuge (86%) but the protection rate was lower (56%) although not significant. Concerning the brands of nets used, both standard pyrethroid-only and PBO-based nets from the mass campaign of 2016–2017 were found in these villages. The Proportion of PBO-based nets was significantly higher in Mayuge (69%) compared to Busia (17%) (Table 1).

### 2.2. Species Composition

The predominant malaria vector in both sites from indoor collection using electric aspirators was the member of the *An. funestus* s.l group followed by *An. gambiae* s.l (Table S2). The density of mosquitoes was higher in Mayuge (1200 for four (4) days of collection) compared to Busia (900 after seven (7) days of collection) despite the high proportion of PBO-based net used in this village. The oviposition rate was the same in both sites for *An. funestus* and *An. gambiae.*

Molecular identification of 99 F0 *An. funestus* s.l mosquitoes (60 oviposited and 39 non-oviposited) from Mayuge by cocktail PCR revealed that 94% (93/99) were *An. funestus s.s*. Three *An. rivolurum* (3%) were detected only among the oviposited females whereas 3 (3%) *An. leesoni* was detected only among the non-oviposited females. All the 110 *An. funestus* s.l (79 oviposited and 31 non-oviposited) analysed in Busia were *An. funestus s.s*. All these results confirm that *An. funestus s.s*. is the predominant malaria vector in both sites at the time of collection.

From the 17 *An. gambiae* s.l successfully identified in Mayuge using SINE PCR, 14 (82.3%) were *An. gambiae* whereas the remaining 3 (17.7%) were *An. arabiensis*. In Busia, out of the 36 oviposited *An. gambiae* s.l PCR tested, a majority was *An. gambiae* s.s at 94.4% (34/36) whereas 5.6% (2/36) were *An. arabiensis*.

### 2.3. Plasmodium Sporozoite Infection Rate

The *Plasmodium* sporozoite-infection rate in oviposited *An. funestus* s.s. from Busia was 3.7% (3/79) and 6% (2/32) in non-oviposited females. In Mayuge, 6.8% (4/59) oviposited *An. funestus* s.s. were infected with *Plasmodium* compared to 8.3% (3/36) in non oviposited females. All the *An. funestus* s.s.were infected only with *P. falciparum.* In *An. gambiae* s.l. from Busia, 11% (3/36) of oviposited females were infected with *P. falciparum* sporozoites and one (01) infected with *P. ovale/vivax/malariae* (OVM+) (2.7%, 1/36). In Mayuge, 6% *An. gambiae* (1/17) were infected with *P. falciparum.*

### 2.4. Insecticide Susceptibility Assays

#### 2.4.1. Bioassays with the Discriminating Concentration 1× (DC)

**Figure 1:**
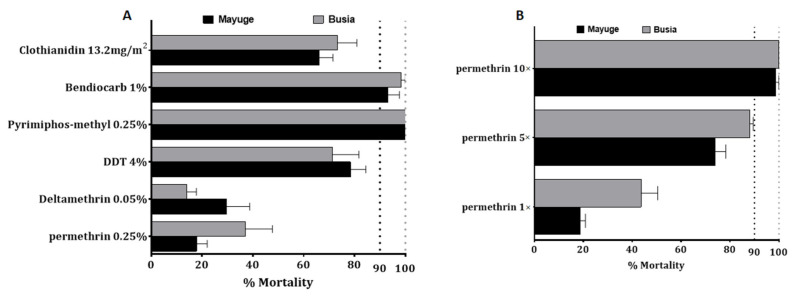
Susceptibility profile to main insecticides of *An. funestus* and *An. gambiae* s.l populations from Busia and Mayuge: (**A**) susceptibility profile of *An. funestus*; (**B**) Susceptibility profile and intensity of *An. gambiae* s.l. Error bars represent standard error of the mean (SEM).

F_1_ progeny from field-collected females *An. funestus* s.s. in both locations showed an extremely high resistance to permethrin and deltamethrin (Figure 1A). F_1_ females from Mayuge showed 17.5 ± 4.2% and 29.7 ± 9.08% mortality 24 h after exposure to permethrin 1× and deltamethrin 1× (DC) respectively (Figure 1A). In *An funestus* s.s from Busia, mortality rates of 37.5 ± 10.5% and 14.08 ± 3.7% were recorded 24 h post-exposure to both pyrethroids tested using the DC (permethrin 1× and deltamethrin 1×) (Figure 1A) while F_1_ females *An. gambiae* s.l showed 43.6 ± 6.8% and 18.7 ± 2.1% mortality to permethrin 1× in Busia and Mayuge respectively (Figure 1B). F_1_ females from both sites presented probable resistance to carbamate, bendiocarb 1× with mortalities of 93.2 ± 4.4%, and 98.3 ± 1.7% in Mayuge and Busia respectively (Figure 1A). Resistance was also noted for the organochlorine, dichlorodiphényltrichloroéthane (DDT) in both sites with mortalities of 78.4 ± 6.01% and 17.2 ± 10.5% in Mayuge and Busia respectively (Figure 1A). However, full susceptibility was observed with the organophosphate, pyrimiphos-methyl 1× with a 100% mortality rate. Mortality rates when exposed to the new insecticide clothianidin 24 h post-exposure were 66.05 ± 5.5% and 73.2 ± 7.7% for Mayuge and Busia respectively (Figure 1A). However, a full susceptibility was observed for this insecticide 72 h post-exposure in both sites with a mortality rate of 100%.

#### 2.4.2. Bioassays with Pyrethroid 5× and 10× DC

**Figure 2:**
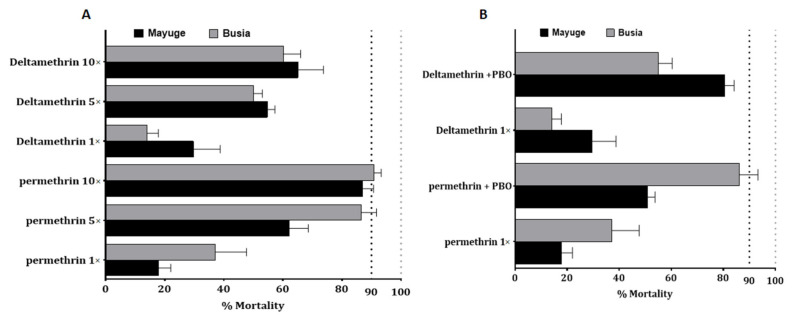
Resistance intensity and synergist assay of *An. funestus* populations from Busia and Mayuge: (**A**) Determination of resistance intensity with 5× and 10× the diagnostic concentrations of permethrin and deltamethrin. Results are average of percentage mortalities ± SEM; (**B**) Effect of pre-exposure to synergist PBO against permethrin and deltamethrin. Results are average of percentage mortalities from four replicates each ± SEM.

Because of the high level of resistance observed to pyrethroids, intensity bioassays were carried out with 5× DC and 10× DC of permethrin (3.75% and 7.5%) and deltamethrin (0.25% and 0.5%). *An funestus* s.s from Mayuge exhibited a mortality rate of 62.4 ± 6.4% and 86.9 ± 3.7% to permethrin 5× and 10× respectively (Figure 2A) pointing a high intensity of resistance to permethrin in this locality. However, a significant increased mortality of *An. funestus* s.s. was observed from permethrin 1× to 5× (χ^2^ = 40.6, *p* ˂ 0.0001) and 10× (χ^2^ = 95.7, *p* ˂ 0.0001). A mortality of 54.7 ± 2.6% and 65.1 ± 8.6% was observed after exposure to deltamethrin 5× and 10× respectively indicating also a high intensity of resistance to deltamethrin in Mayuge (Figure 2A). Similar observations were made in Busia with mortality rates of 86.4 ± 5.2% and 90.9 ± 2.4% (for permethrin 5× and 10×) and 50.02 ± 3.01% and 60.1 ± 5.9% (for deltamethrin 5× and 10×) (Figure 2B). However, *An. gambiae* s.l displayed a moderate intensity of resistance to permethrin in both locations with mortality of 88.06 ± 1.4% and 100% to permethrin 5× and 10× respectively in Busia and 73.9 ± 4.3% for permethrin 5× and 98.5 ± 1.4% for permethrin 10× in Mayuge (Figure 1B).

#### 2.4.3. Bioassays with the Synergist PBO

Bioassay using PBO as a synergist revealed a partial recovery of susceptibility to permethrin and deltamethrin. In Mayuge the mortality with permethrin + PBO was 50.9 ± 2.3% *versus* 17.8 ± 4.2% for permethrin without PBO pre-exposure (χ^2^ = 25.7 *p* < 0.0001). The same pattern was observed for deltamethrin: PBO pre-exposure 80.6 ± 3.5% vs. 29.7 ± 9.08% for no PBO pre-exposure (χ^2^ = 51.2 *p* < 0.0001) (Figure 2B). Synergist bioassay in Busia also revealed partial recovery of susceptibility to both pyrethroids [permethrin: PBO pre-exposure 86.2 ± 7.1% *versus* 37.1 ± 10.5% mortality without PBO pre-exposure (χ^2^ = 30.9 *p* < 0.0001); deltamethrin: PBO pre-exposure 55.07 ± 5.3% vs. 14.08 ± 3.7% mortality for no PBO pre-exposure (χ^2^ = 24.1 *p* < 0.0001)] (Figure 2B). These results show that cytochrome P450s are playing only a partial role in the escalation of resistance observed in Busia and Mayuge.

### 2.5. Bioefficacy of Insecticide-Treated Bed Nets

**Figure 3:**
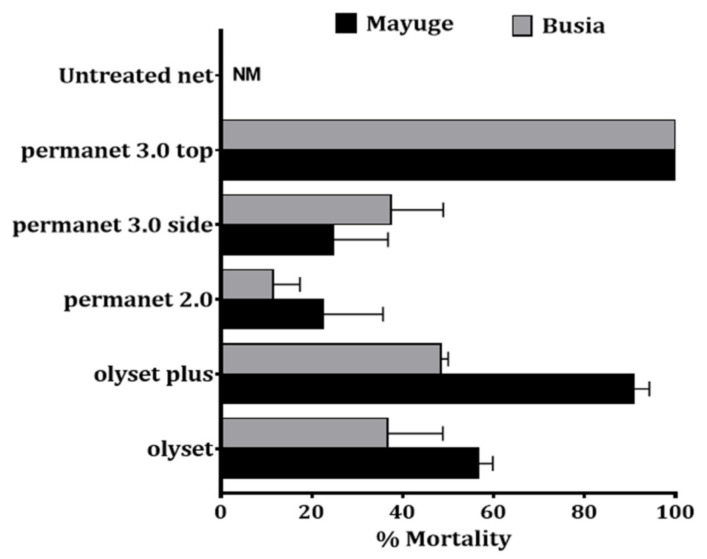
Bio-efficacy of different commercial LLINs against *An. funestus* in Busia and Mayuge. Results of cone bioassays with PermaNet^®^3.0 (side and roof), PermaNet^®^2.0, Olyset^®^Plus and Olyset^®^Net. Results are average of percentage mortalities ± SEM of five replicates. NM = no mortality.

To evaluate the impact of super resistance on the efficacy of control tools, cone assays were conducted with various LLINs. In both localities, low efficacy of standard pyrethroid-only nets (Olyset and PermaNet 2.0) was observed against *An. funestus* s.s.: Olyset: 56.7 ± 3.1% and 36.7 ± 12.1% mortality for Busia and Mayuge respectively; PermaNet 2.0: 22.5 ± 13.1% and 11.4 ± 6.9% mortality for Mayuge and Busia respectively. However, PBO-based nets (Olyset Plus, and PermaNet 3.0) showed an increased efficacy (Olyset Plus: 90.8 ± 3.5% mortality; PermaNet 3.0-side: 24.7 ± 12.0%, PermaNet 3.0-roof: 100.0 ± 0.0%) in Busia (Figure 3). In contrast, in Mayuge increased susceptibility was noted only for PermaNet 3.0-roof (100.0 ± 0.0% mortality). A significantly reduced efficacy of the PBO-net Olyset plus (48.5 ± 1.5% mortality) was observed in this site confirming the very low mortality recorded with Permethrin + PBO in this site. The mortality with PermaNet 3.0 side did not differ significantly to that of PermaNet 2.0 (24.7 ± 12% vs. 22.5 ± 12% in Mayuge (χ^2^ = 0.07; *p* = 0.8) but in Busia (37.4 ± 11.5% vs. 11.4 ± 6.9%; χ^2^ = 9.07; *p* = 0.003)) indicating the high intensity of resistance in these locations particularly in Mayuge. Pyrethroid-only and PBO-nets used in this study induced total mortality against the control Kisumu susceptible *An. gambiae* mosquitoes (Figure 3).

### 2.6. Distribution of Insecticide Resistance Markers in An. funestus

**Figure 4:**
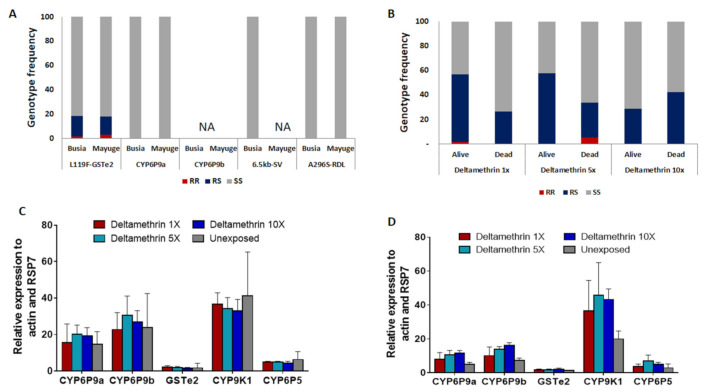
Analysis of the molecular basis of the escalation of pyrethroid resistance in *An. funestus*: Distribution of the genotypes for key resistance markers in F0 female *An. funestus* from Eastern Uganda including *CYP6P9a*, *CYP6P9b*, L119F-GSTe2, and A296S-RDL showing low frequency of resistant genotypes (**A**) and Distribution of the genotypes at the L119F-GSTe2 locus between mosquitoes alive and dead after exposition to deltamethrin 1×, 5× and 10× (**B**). Differential gene expression of the P450 genes *CYP6P9a*, *CYP6P9b, CYP9K1,* and *CYP6P5* and the Gluthatione S-tranferase *GSTe2* in *An. funestus* from Mayuge (**C**) and Busia (**D**), error bars represent standard error of the mean.

In total, 86 females *An. funestus* s.s (50 oviposited and 36 non-oviposited) from Mayuge and 82 (50 oviposited and 32 non-oviposited) from Busia were genotyped for different resistance markers. In Busia, out of 40 oviposited females which successfully amplified for the L119F mutation, only one (1) were homozygote resistant, six (06) were heterozygotes and 33 were homozygote susceptibles corresponding to the frequency of 10%. In non-oviposited, the frequency of this mutation was 9.6% (0 RR, 5 RS, and 21 SS) (Figure 4A). In Mayuge, the frequency of this mutation was 11.5% (0RR, 9RS, and 30SS) in oviposited females and 10.3% (2RR, 2RS, and 29 SS) in non-oviposited females (Figure 4A). A significant association was found in the ability of mosquitoes with the 119F resistant allele to survive exposure to deltamethrin 1× (OR = 2.7; confidence interval (CI) = 1.3–5.6; *p* = 0.004) but not the 5× (OR = 1.7; confidence interval (CI) = 0.9–3.4; *p* = 0.07) and 10× (OR = 0.5; confidence interval (CI) = 0.3–1.2; *p* = 0.08) (Figure 4B). This shows that the mutation is linked with deltamethrin resistance but not implicated in the escalation of resistance.

The A296S-RDL mutation conferring dieldrin resistance [11] and the CYP6P9a_R allele conferring pyrethroid resistance were completely absent in oviposited and non oviposited females from both locations (Figure 4A). The PCR-RFLP recently designed to detect the resistant allele at the *CYP6P9b* locus and a multiplex PCR designed for the 6.5 kb-sv failed to amplify (Figure 4A).

### 2.7. Distribution of Insecticide Resistance Markers in An. gambiae

The L1014F-KdrW mutation was completely absent in both *An. gambiae* and *An. arabiensis* from Mayuge and Busia. However, the L1014S-KdrE mutation was fixed in *An. gambiae* (100%RR) and completely absent in *An. arabiensis.* The N1575Y-kdr mutation conferring pyrethroid resistance and the G119S-Ace1 mutation conferring carbamate resistance were completely absent in both species in both localities.

### 2.8. Transcriptional Profiling Of Metabolic Resistance Genes in An. funestus s.s.

Transcription analysis of *CYP9K1* and the duplicated P450 genes *CYP6P9a* and *CYP6P9b* known to confer pyrethroid resistance in *An. funestus* [12,13] reveals a high up-regulation of these genes in deltamethrin resistant mosquitoes from both site particularly the *CYP9K1* (fold change, FC = 36.7 ± 10.3; FC = 34.15 ± 6.1; FC = 32.9 ± 6.3; FC = 41.1 ± 24.1 for deltamethrin alive 1×, 5×, 10× and unexposed respectively) and *CYP6P9b* (fold change, FC = 22.7 ± 9.3; FC = 30.4 ± 10.6; FC = 26.8 ± 6.2; FC = 23.7 ± 18.7 deltamethrin alive 1×, 5×, 10× and unexposed respectively) compared to the susceptible FANG strain (*p* < 0.001) (Figure 4C). However, the expression of these genes was not statistically different between mosquitoes alive to DC, 5× DC, and 10× DC of deltamethrin showing that these genes might not play a major role in the increased resistance. The *CYP6P5* and the *GSTe2* (conferring DDT/permethrin resistance in West/Central Africa [14]) were not significantly upregulated in Uganda (Figure 4D).

## 3. Discussion

Worrying cases of high resistance levels to insecticides are emerging in major malaria vectors leading to extensive loss of efficacy of current and probably future vector control tools. Quantifying the resistance intensity and establishing the molecular drivers of such resistance escalation is crucial for designing resistance management strategies and prevent malaria resurgence.

This study revealed a high intensity of resistance to pyrethroids with significantly reduced efficacy of insecticidal treated LLINs including standard nets (Olyset^®^ Net and PermaNet^®^ 2.0) and PBO-based net (Olyset plus) in *An. funestus* s.s. from Eastern Uganda. Similar results were obtained recently in *An. funestus* s.s. from Southern Mozambique revealing a very low efficacy of the two most common commercial LLINs used across Africa, Olyset^®^ Net, and PermaNet^®^ 2.0 [4,8]. The same loss was also reported Malawi (<5% mortality) [15] and in Democratic Republic of Congo (<35% mortality) [3]. This loss in efficacy of bednets in Mayuge and Busia was in line with the results of WHO tube assays, pointing a high intensity of resistance to permethrin and deltamethrin, the insecticides used in these nets. These *An. funestus* populations were resistant to permethrin and deltamethrin at all diagnostic concentrations of 1×, 5×, and 10×. However, the *An. gambiae* s.l populations from both sites are resistant to permethrin at 1× and 5× but not 10×. These results for *An. gambiae* is similar to the observations of Okia et al. (2018) in Tororo [2]. However, this study is the first evidence of resistance escalation in *An. funestus* in Uganda using the 5× and 10× DC of pyrethroids. However, the high level of resistance to the diagnostic dose (1×) of pyrethroids in *An. funestus* is similar to the temporal increase in pyrethroid resistance observed in Eastern Uganda between 2009 and 2013 [16]. Probable resistance was observed to bendiocarb in *An. funestus* population from both locations showing that the carbamate insecticides could not be an alternative to pyrethroid for IRS. Furthermore, the full susceptibility to the organophosphate pyrimiphos-methyl, as observed in many other African countries across the continent, suggests that this insecticide class is the most suitable for IRS against this species and justifies the use of Arctellic for IRS in many districts in Uganda.

This study reported low mortality after exposure to permethrin+PBO and deltamethrin + PBO together with the loss in the efficacy of the new generation of PBO-based nets particularly the Olyset plus. This reduced efficacy of PBO-based tools could be linked to the fact that PBO-net are already in use in the study site and could be contributing to the selection of other resistance mechanisms beyond cytochrome P450s. The ability of mosquitoes to survive exposure to the high intensity of pyrethroids and PBO-based nets in this population is problematic for malaria control programs. These observations are similar to the results obtained with *An. funestus* in Cameroon [17] and *An. coluzzi* in Chad [6] for which no mortality was noticed after exposure to the synergist net Olyset plus. However, the reduced efficacy of PBO nets has not previously been observed in *An. funestus* as higher mortality rates (>80%) have so far been reported when testing PBO-based nets (Olyset Plus) against other pyrethroid-resistant populations like in Malawi [15] and DR Congo [3]. The PermaNet 3.0 Top (containing PBO) showed a higher efficacy with 100% mortality. Nevertheless, the low mortality with PermaNet 3.0 (side) obtained in this study is similar to the results obtained in Cameroon [17], Mozambique [4], and Chad [6] but contrary to the higher mortality rate (88%) observed in DRC [3] suggesting also an overall loss of efficacy of PermaNet 3.0 in Uganda. The causes of such high resistance could be associated with the scale-up of LLINs distribution across the country as PB0-nets were present in both locations. The massive use of pyrethroids in agriculture in Uganda could be another factor in selecting for resistance in malaria vectors [2].

Genotyping of resistant markers showed the absence of CYP6P9a_R resistant allele, the predominant pyrethroids resistance marker in southern Africa [18] showing that other mechanisms are responsible for pyrethroids resistance in these *An. funestus* mosquitoes. Furthermore, the moderate resistance to DDT supports the low frequency of L119F-*GSTe2* in these populations compared to West and Central Africa. The *CYP9K1* was the most over-expressed gene in deltamethrin-resistant mosquitoes from both localities showing that this gene is likely the main driver of pyrethroid resistance as previously shown for the ortholog of this gene in *An. gambiae* [19,20]. However, the expression of this gene did not vary significantly between resistant mosquitoes at 10× and 5× compared to 1× showing that other mechanisms are playing a major role in the escalation of resistance in these *An. funestus* populations. The duplicated *CYP6P9a* and *CYP6P9b* were also overexpressed but at a reduced level compared to southern Africa (Mozambique and Malawi) where these genes are highly over-expressed [5,13,14]. The *GSTe2* gene conferring DDT resistance in West/Central Africa was also significantly over-expressed in both localities but at very low level compared to Benin with more than 80 fold-change observed in resistant mosquitoes [14]. Nevertheless, the expression level of the gene was higher than the four (4)-fold change observed in Uganda in 2014 [16].

The sporozoite infection rates of *An. funestus* and *An. gambiae* populations of Busia and Mayuge is similar to the rates generally reported in this country [16,21] and across the continent. However, the infection rate observed in these two locations was higher compared to the areas of the country where IRS have been implemented such as Tororo where a recent study showed a significant reduction in *plasmodium* infection rate [22]. Therefore, the high infection rates of *An. gambiae* and *An. funestus* in Busia and Mayuge highlights the need of giving further attention to both *species* when it comes to vector control. This observation also may indicate that IRS with organophosphate could be the most appropriate control intervention compared to LLINS (impregnated with pyrethroid mainly).

## 4. Materials and Methods

### 4.1. Mosquito Collection

Indoor resting and blood-fed female *Anopheles* mosquitoes were collected in two districts in Eastern Uganda. Initially, four districts were selected (Tororo, Busia, Jinja, and Mayuge), and due to the low density of mosquitoes, collections were performed mainly in two districts (Busia and Mayuge). In each district, one village was chosen: Bumanji (0°27′08.4″ N, 34°06′38.1″ E) in Busia and Bubbalya (0°23′10.8″ N, 33°37′16.5″ E) in Mayuge. The collection was performed for 7 days in Busia and 4 consecutive days in Mayuge in February 2020. Mosquitoes were collected using electric aspirators after recording the information on the types of houses and the bed-nets use.

*Anopheles* females mosquitoes collected were morphologically identified as belonging to *An. funestus* group or *An. gambiae* s.l complex according to morphological keys [23]. These mosquitoes were kept in carton cups and fed with sugar until they became fully gravid before forced egg-laying in 1.5 mL micro-centrifuge tubes and larvae reared to adults as previously described [24].

### 4.2. Molecular Identification of Field-Collected Females

Oviposited and non-oviposited females *An. funestus* s.l. and *An. gambiae* s.l were cutted into head/thorax and abdomen for genomic DNA (gDNA) extraction using the Livak method [25]. A cocktail polymerase chain reaction (PCR) was used for species identification of *An. funestus* members as previously described [26]. The SINE PCR assay [27] was used for the identification of the *An. gambiae* species.

### 4.3. Plasmodium Infection Rates

*Plasmodium* sporozoite infection rate was assessed using TaqMan assay in heads plus thoraxes gDNA from F_0_ *An. funestus* s.s. and *An. gambiae* s.l as previously described [28].

### 4.4. Insecticide Susceptibility Assays

The susceptibility pattern of both *An. funestus* and *An gambiae* s.l to various insecticides were assessed using the WHO-tube bioassays [10]. *An. funestus* s.s. mosquitoes from both locations were tested to the pyrethroids type I permethrin (0.75%) and type II deltamethrin (0.05%), the organochlorine DDT (4%), the carbamate bendiocarb (0.1%), and the organophosphate pyrimiphos-methyl (0.25%). In addition, the new insecticide, clothianidin, was also tested using the SumiShield formulated product at a dosage of 13.2 mg/m^2^. Because of the low number of *An. gambiae* s.l collected, tests were performed only for permethrin. All the tests were performed at standard insectary conditions of 25 ± 1 ° C temperature and 70–80% relative humidity. For each test, four replicates of 20–25 F_1_ female mosquitoes of 2–5 day-old were exposed to insecticide-impregnated papers for 1 h. After the exposure, mosquitoes were transferred to a holding tube provided with cotton soaked in a 10% sugar solution. Mortality was determined 24 h later. Control tubes with non-impregnated papers were performed for each bioassay.

Based on the results of resistance status with 1× (discriminating concentration (DC)) of pyrethroid (permethrin and deltamethrin), intensity bioassays were carried out with 5× DC and 10× DC of these insecticides. The intensity bioassays with 5× and 10× DC were performed following the WHO 2016 test procedure [10]. Synergist assays with piperonyl butoxide (PBO; an inhibitor of cytochrome P450s) were performed for the potential involvement of P450′s genes.

### 4.5. Insecticide-Treated Bed Nets Efficacy Assays

The effectiveness of the LLINs was estimated following the WHO guidelines for cone bioassays [29]. The nets tested were Olyset^®^ Net (permethrin 2%) and Olyset^®^ Plus net roof (permethrin 2% plus PBO 1% in the roof) for the PBO-nets; PermaNet^®^ 2.0 (deltamethrin 0.18%) and PermaNet^®^ 3.0 side (deltamethrin 0.28%) for the standard nets. An untreated mosquito net was used as a control. Five replicates of ten F_1_ 2–5 days old females were placed in plastic cones enclosed with the mosquito net for 3 min. Mosquitoes were then transferred in small holding paper cups with cotton soaked in a 10% sugar solution. Mortality was determined 24 h later.

### 4.6. Genotyping of Resistance Markers in An. funestus s.s.

The presence of resistance markers including L119F-GSTe2 (DDT/permethrin), CYP6P9a, CYP6P9b, 6.5kb-SV (pyrethroid), and A296S-RDL (dieldrin) was assessed in *An. funestus* s.s. The A296S-RDL mutations were genotyped using TaqMan assays as previously described [15], an allele-specific PCR (AS-PCR) was used to genotype the L119F-GSTe2 [30,31] whereas the presence of the CYP6P9a/b_R allele was assessed using PCR-RFLP assays recently designed [18,32]. Finally, the 6.5kb-SV was genotyped using a multiplex PCR [33].

Furthermore, the L119F mutation was genotyped in deltamethrin alive and dead (1×, 5×, and 10×) to assess a potential association between this marker and the aggravation of deltamethrin resistance.

### 4.7. Genotyping of Resistance Markers in An. gambiae.s.l

TaqMan assays with two labeled fluorochromes probes FAM and HEX were used to screen for the L1014F and L1014S-*kdr* [28] and the N1575Y [28] mutation associated with DDT and pyrethroid resistance in *An. gambiae* s.l. Further, the G119S-*ace*-*1* responsible for organophosphate and carbamate resistance in *An. gambiae* s.l. was also genotyped in Mayuge and Busia mosquitoes using TaqMan assays.

### 4.8. Transcription Profile of Resistance Genes in An. funestus s.s.

The transcription patterns of *CYP9K1*, *CYP6P5*, *CYP6P9a*, *CYP6P9b*, and *GSTe2*) major pyrethroid resistance genes [34] were assessed by a quantitative reverse transcription PCR (qRT-PCR) in deltamethrin 1× alive, 5× alive, 10× alive and unexposed mosquitoes relatively to the susceptible strain FANG. Total RNA was extracted from 3 batches of 10 mosquitoes for each group and similarly from the susceptible laboratory strain FANG, as previously described [12]. The relative expression was calculated individually according to the 2^−ΔΔCT^ method [35] and compared between different groups.

## 5. Conclusions

The extremely high intensity of resistance coupled with the loss of efficacy of impregnated bed nets against *An. funestus* s.s. in Uganda represents a serious threat for vector control in Uganda. The common resistant P450-based mechanisms were not found to play a role in the escalation of resistance highlighting the urgent need to investigate the causes such super-resistance and to monitor the spread of such operationally significant resistance in other mosquito populations and assess its impact on malaria transmission.

## Figures and Tables

**Table 1:** Household indices and brand nets from the collection site.

Household Indices	Busia	Mayuge
Number of houses	25	35
Houses with bednet	23	30
Bed net coverage	92% (CI: 75–97)	86% (CI: 71–94)
Number of inhabitant	89	177
N° of inhabitants Protected	53	99
Protection rate	60% (CI: 49–69)	56% (CI: 43–57)
**Brands of Nets Used**	**Busia**	**Mayuge**
Olyset	7 (23%)	6 (12%)
Permanet 2.0	16 (53%)	8 (16%)
Permanet 3.0	5 (17%)	34 (69%)
Unknown	0 (0%)	1 (2%)
**Total**	30 (100%)	49 (100%)

CI: confidence interval.

## Data Availability

All the data is present in the manuscript and supplemental files.

## Supplementary Materials

The following are available online at https://www.mdpi.com/article/10.3390/pathogens10040415/s1, Table S1: Type of houses in the collection sites Table S2: Number of mosquitoes collected and the oviposition rate per site.
