# Supplementary material for: Pyrethroid Resistance Aggravation in Ugandan Malaria Vectors Is Reducing Bednet Efficacy
Source: Pathogens. 2021 Apr 1;10(4):415. doi: 10.3390/pathogens10040415 (PMC8065452; doi:10.3390/pathogens10040415)
Supplement: Supplementary file 1 [file pathogens-10-00415-s001.pdf]

Table 1. Type of houses in the collection sites.

Site	Type of house				
	Circular hut with thatched roof	Rectangular thatched roof	Rectangular with corrugated iron roof		Total
Busia	6 (24%)	6 (24%)	13 (52%)		**25 (100%)**
Mayuge	4 (11.4%)	17 (48.6%)	14 (40%)		**35 (100%)**
	Wall surface				
	Brick	Cement	Mud	painted Cement	Total
Busia	0 (0%)	3 (12%)	22 (88%)	0 (0%)	**25 (100%)**
Mayuge	8 (22.9%)	0 (0%)	26 (74.3%)	1 (2.8%)	**35 (100%)**

Table 2. Number of mosquitoes collected and the oviposition rate per site.

Collection sites	Total collected	Forced to lay		Oviposition	
	Anopheles spp	*An. funestus*	*An. gambiae*	*An. funestus*	*An. gambiae*
Busia	~ 900	586	163	342 (58%)	58 (35%)
Mayuge	~ 1200	1050	78	662 (63%)	26 (33%)
**Total**	**2100**	**1636**	**241**	**1004 (61%)**	**84 (34%)**
